# A Comparative Study of Flavonoids and Carotenoids Revealed Metabolite Responses for Various Flower Colorations Between *Nicotiana tabacum* L. and *Nicotiana rustica* L.

**DOI:** 10.3389/fpls.2022.828042

**Published:** 2022-04-25

**Authors:** Qinzhi Xiao, Yueyi Zhu, Guoxian Cui, Xianwen Zhang, Risheng Hu, Zhengyu Deng, Lei Lei, Liwen Wu, Lei Mei

**Affiliations:** ^1^Yongzhou Tobacco Monopoly Bureau of Hunan, Yongzhou, China; ^2^College of Agronomy, Hunan Agricultural University, Changsha, China; ^3^College of Agriculture and Biotechnology, Zhejiang University, Hangzhou, China; ^4^Institute of Virology and Biotechnology, Zhejiang Academy of Agricultural Sciences, Hangzhou, China; ^5^College of Plant Science and Technology, Huazhong Agricultural University, Wuhan, China; ^6^College of Bioscience and Technology, Hubei Minzu University, Enshi, China; ^7^Department of Plant Sciences, University of Cambridge, Cambridge, United Kingdom

**Keywords:** floral color, *Nicotiana tabacum* L., *Nicotiana rustica* L., flavonoid, carotenoid

## Abstract

Tobacco is a model plant for studying flower coloration. Flavonoids and carotenoids were reported to contribute to the flower color in many plants. We investigated the mechanism underlying flower color formation in tobacco by comparing the profiling flavonoids and carotenoids between various species *Nicotiana tabacum* L. and *Nicotiana rustica* L., as their flowers commonly presented red (pink) and yellow (orange), respectively. The metabolomes were conducted by UPLC–ESI–MS/MS system. The main findings were as follows: (1) A total of 31 flavonoids and 36 carotenoids were identified in all four cultivars involved in *N. tabacum* and *N. rustica*. (2) Flavonoids and carotenoids tended to concentrate in the red flowers (*N. tabacum*) and yellow flowers (*N. rustica*), respectively. (3) About eight flavonoids and 12 carotenoids were primarily screened out for metabolic biomarkers, such as the robust biomarker involving kaempferol-3-*o*-rut, quercetin-glu, rutin, lutein, and β-carotene. This is the first research of systematic metabolome involving both flavonoids and carotenoids in tobacco flower coloration. The metabolic mechanism concluded that flavonoids and carotenoids mainly contributed to red (pink) and yellow (orange) colors of the tobacco flowers, respectively. Our finding will provide essential insights into characterizing species and modifying flower color in tobacco breeding through genetic improvement or regulation of featured metabolic synthesis.

## Introduction

As one of the five major genera of the family Solanaceae, the genus *Nicotiana* consists of 64 species, of which 44 and 20 species are considered to be originated in the America and Oceania continent, respectively (Goodspeed, 1954). According to the morphologies, chromosomal number, cross-ability relationships, and features in interspecific hybrids, the species under genus *Nicotiana* were further classified into three sub-genera *tabacum, rustica*, and *petunioides*, among which *N. tabacum* and *N. rustica* were the main kinds of cultivars (Siva Raju et al., 2008; Huang et al., 2021). Generally, the red (pink) and yellow (orange) flowers were commonly phenotyped in *N. tabacum* and *N. rustica*, respectively. Currently, the mechanisms underlying the coloration in tobacco are not clear. Floral color can protect floral organs and attract pollinators. Notably, it was a crucial trait in resolving the evolutionary relationships between species. The coloration was determined by the types and amounts of pigments, which were also influenced by the internal or surface tissue structure of the petals (Zhao and Tao, 2015). The pigment components of colorful flowers have been studied for over 150 years, since the mid-19th century. Carotenoids and flavonoids are the two primary types of substances that give color to flowers (Maoka, 2020; Mekapogu et al., 2020).

Plants produce more than 1,000,000 metabolites in prediction (Afendi et al., 2012). Flavonoids are one of the major groups of specialized metabolites and by far the largest class of polyphenols. Currently, flavonoids are estimated to include more than 8,000 compounds (Yonekura-Sakakibara et al., 2019; Wen et al., 2020). The flavonoids process a common backbone of diphenylpropane (C6–C3–C6), where the two aromatic rings are linked *via* a three-carbon chain (Wen et al., 2020). One of the rings is originated from resorcinol or phloroglucinol produced by the acetate pathway (Perez et al., 2020). Correspondingly, another ring is formed from the shikimate pathway (Saito et al., 2013). Generally, flavonoids are divided into six subclasses, that is, flavones, isoflavones, flavonols, flavonols, flavanones, and anthocyanidins (Harbone, 1993). Flavonoids are the most critical pigments in plants, which exhibit the broadest spectrum of colors.

Carotenoids are a group of natural tetraterpenoid pigments synthesized by plants, bacteria, algae, and fungi (Fuentes et al., 2012; Avalos and Carmen Limón, 2015; Sun et al., 2018). Thus far, more than 1,100 natural carotenoids have been reported (Yabuzaki, 2017; Quian-Ulloa and Stange, 2021). In plants, carotenoids play vital roles in photoprotection and photosynthesis, as well as the precursors for phytohormones such as abscisic acids and strigolactones (Nambara and Marion-Poll, 2005; Domonkos et al., 2013; Niyogi and Truong, 2013; Al-Babili and Bouwmeester, 2015; Hashimoto et al., 2016). Furthermore, carotenoids also serve as signaling molecules in plant developments and respond to environments (Havaux, 2014; Tian, 2015; Hou et al., 2016). According to the structures, carotenoids can be divided into two classes, namely, carotenes (hydrocarbons without oxygen) and xanthophylls (hydrocarbons with oxygen). As the derivatives of tetraterpenes, carotenoids are molecules of a C40 polyene backbone with an ionone ring at the end. Based on this structure, carotenoids can absorb visible light of short wavelengths ranging from 400 to 550 nm (violet to green light). The number and properties of the double bonds determined the wavelength of light absorbed. Hence, the compounds are colored yellow, orange, or red.

Biomarkers are defined to predict phenotypical properties while these features are not apparent, which are valuable tools for life research (Meyer et al., 2007; Collard and Mackill, 2008; Geifman-Holtzman and Ober, 2008). Generally, the conventional biomarkers are defined as genetic markers, which are comprehensively applied to identify specific genetic lines with positive traits. However, to some genetic markers in plants, it's challenging for species with complex polyploid genomes. Metabolic profiles in a biological system are the endpoints in omics involving genome, transcriptome, and proteome (Khamis et al., 2017). Hence, the exploration of new metabolic biomarkers would provide a new approach to identifying such species due to not being based on genomics. Significantly, some tobaccos, such as allotetraploid *N. tabacum*, have complicated genomes.

Tobacco was considered a vital model plant for studying flower coloration. To date, the previous studies of flower coloration in tobacco commonly focused on and limited to a few genes involving anthocyanin biosynthesis (Li et al., 2017; Park et al., 2020), or transcriptome and metabolome engaged in the same *Nicotiana* species (Jiao et al., 2020). The color of flowers results from the combined action of various metabolites, while the close and same color of flowers may be determined by different metabolic components (Zhao and Tao, 2015). However, red (pink) and yellow (orange) are the two most common flower colors in tobacco. They are also the typical flower colors of different species, such as *N. tabacum* and *N. rustica*, respectively. Hence, systematically identifying the metabolites would provide insights into the mechanisms underlying the formation of differentiated flower color. Moreover, the exploration of related metabolic biomarkers would offer alternative tools for the identification of tobacco species.

## Materials and Methods

### Plant Materials and Sampling

Species *N. tabacum*, including two cultivars No-2146 and Xiangyan-3, were employed in the experiments, and they presented red (pink) flowers. For convenience, the two cultivars were named RFA and RFB in this study, respectively. Parallelly, species *N. rustica*, involving two cultivars Laolaihuang and Xiaoyelanhua, showed both yellow (orange) flowers and called YFA and YFB in this study. Additionally, No-2146 were cultivated by the Hunan Tobacco Monopoly Bureau as lineage “{[(G28 × Coker176) × (K326 × Gexin-3)] × K326} × K32.” G28, Coker176, K326, Gexin-3, and K32 are common *N. tabacum* species in China. Xiangyan-3 was the progeny of K394 × Yunyan-87. Cultivars Laolaihuang and Xiaoyelanhua were *N. rustica* species and commonly used in many laboratories. The tobacco seeds that were employed in this study originated from the Yongzhou Tobacco Germplasm Resource Database (YTGRD, Yongzhou 425000, P. R. China). The plants were grown in a glasshouse at the Agricultural Experiment Station, Zhejiang University. Seeds of the four varieties were germinated in glass Petri dishes before wet filter papers were paved. The dishes containing seeds were transferred to the program ray radiation incubator until the green shoots appeared. The conditions involved light intensity of 100 mol/m^2^/s, light/dark 14 h/10 h, 22°C, and humidity 60%. Consequently, the germinated seeds transfer onto the pots filled with quartz sand. The pots were placed in a greenhouse under 300 mol/m^2^/s, light/dark 14 h/10 h, 28°C, and humidity 60%. The pots were watered every 3 days and fertilized every 9 days. The liquid fertilizer was one-fourth of the nutrient solution concentration (Bovet et al., 2006). The redundant seedlings were removed and retained one seedling in each pot. On the 20th day after the first blooming flower appeared, the petals of all the four varieties were collected and kept in a −80°C refrigerator for use. Three biological replicates were taken from individual plants within every variety.

### Extraction and Analysis of the Flavonoids and Carotenoids

For extraction of flavonoids, the freeze-dried samples were ground into powder by a mixer mill (MM,400, Retsch, Germany) for 2 min at 30 Hz. Next, the 100 mg powdered sample was mixed with 1.0 ml methanol/water/hydrochloric acid (500:500:1, v/v/v), and then overnight at 4°C. Consequently, ultrasound was applied to the extract for 8–10 min and centrifuged at 13,000 *g* at 4°C for 8 min. The supernatants were collected and filtrated with a membrane filter (0.22 μm pore size, Anpel, Shanghai, China). The final filtrate was kept for LC–MS/MS analysis. For extraction of carotenoids, the freeze-dried samples were homogenized and powdered in the same mill. A 100 mg powdered sample was extracted with 1 ml of compound solution of hexane/acetone/ethanol (2:2:1, v/v/v) and extra 0.1% butylated hydroxytoluene. Then the internal standards were added. The extract was vortexed for 15 min at room temperature and consequently centrifuged at 13,000 g at 4°C for 8 min. The supernatants were collected, and the residue was re-extracted at the same conditions. The final supernatant liquid was evaporated under a nitrogen gas stream. Next, the dried residues were dissolved by 200 μl of compound solution of (ethyl nitrile/methyl alcohol)/methyl tert-butyl ether (17(3/1)/3, v/v/v). Finally, the solution was filtered by more than 0.22-μm filter for the upcoming analysis.

### Detection of the Flavonoids and Carotenoids

Flavonoids and carotenoids were detected based on the platform UPLC–ESI–MS/MS system (UPLC'ExionLC AD'https://sciex.com.cn/; MS'Applied Biosystems 6500 Triple Quadrupole, https://sciex.com.cn/), and UPLC–APCI–MS/MS system (UPLC'ExionLC AD https://sciex.com.cn/; MS, Applied Biosystems 6500 Triple Quadrupole, https://sciex.com.cn/), respectively. The analytical conditions of flavonoids were shown as HPLC: column, Waters ACQUITY UPLC HSS T3 C18 (1.8 μm, 100 × 2.1 mm); Solvent A, water, 0.04% acetic acid; Solvent B (acetonitrile, 0.04% acetic acid); Gradient program, 100:0, 5:95, 5:95, 95:5, and 95:5 of V(A)/V(B) at 0, 11.0, 12.0, 12.1, and 15.0 min, respectively; Flow rate, 0.40 ml/min; Temperature, 40 °C; Injection volume, 5 μl. Parallelly, the analytical conditions of carotenoids were followed, LC: column, YMC C30 (3 μm, 2.0 mm × 100 mm); Solvent A, methanol acetonitrile (1/3, v/v) with 0.1% formic acid and 0.01% butylated hydroxytoluene (BHT); Solvent B, methyl tert-butyl ether with 0.01% BHT; Gradient program, 0%, 70%, 95%, 0% Solvent B were used for 3, 2, 4, and 1 min, respectively. Flow rate, 0.8 ml/min; Temperature, 28°C; Injection volume, 2 μl. All 12 samples' reproducibility was evaluated *via* principal component analysis (PCA). Unsupervised PCA was performed by R (www.r-project.org). The data were unit variance scaled before unsupervised PCA.

### Hierarchical Cluster Analysis and Screening of Metabolic Biomarkers

The hierarchical cluster analysis in terms of metabolites and samples was exhibited as heatmaps combined with dendrograms, which were conducted by *R* package heatmap involving algorithm pheatmap (m, scale = “row”) and the normalized signal intensities of metabolites, as unit variance scaling are visualized as a color spectrum. In the experiments, the metabolic biomarkers were screened out based on the following criteria: (1) In the same sample, the content of metabolic biomarker is at least two times higher than that of other metabolites; therefore, the metabolic biomarker can be distinguished from other metabolites in the same sample; (2) For metabolic biomarkers, the highest content value was equal or more than two times of lowest content value among the differentiated groups aimed at, that is, the contents of metabolic biomarkers varied significantly among different samples.

### Differential Metabolites Screened and KEGG Annotation and Enrichment Analysis

Based on the orthogonal projections to latent structures discrimination analysis (OPLS-DA) (Thevenot et al., 2015), the variable importance in projection (VIP) of the obtained multivariate analysis of OPLS-DA, was able to preliminarily screen out metabolites that differ in different varieties or tissues. At the same time, the Fold_Change of the univariate analysis further screened out the difference in metabolism. Combining Fold_Change and the VIP value of the OPLS-DA model, differential metabolites were filtered. Filter criteria are as follows: (1) Select metabolites with Fold Change ≥ 2 and Fold Change ≤ 0.5. If the difference of metabolites is more than 2 or less than 0.5 times between the control and experimental groups, the difference is considered significant; (2) Select metabolites with VIP≥1, first. The VIP value represents the influence strength of the corresponding metabolite between groups, and it is generally considered that the metabolites with VIP ≥ 1 are significantly different. Schematics of flavonoids and carotenoid synthesis were constructed according to literature involving plants (Liu et al., 2020). The identified metabolites were mapped onto the two pathways and checked with the KEGG pathway database (http://www.kegg.jp/kegg/pathway.html) (Kanehisa and Goto, 2000).

## Results

### Morphological Characterization of Species and the Quality Assessment of Metabolomes

**Figure 1:**
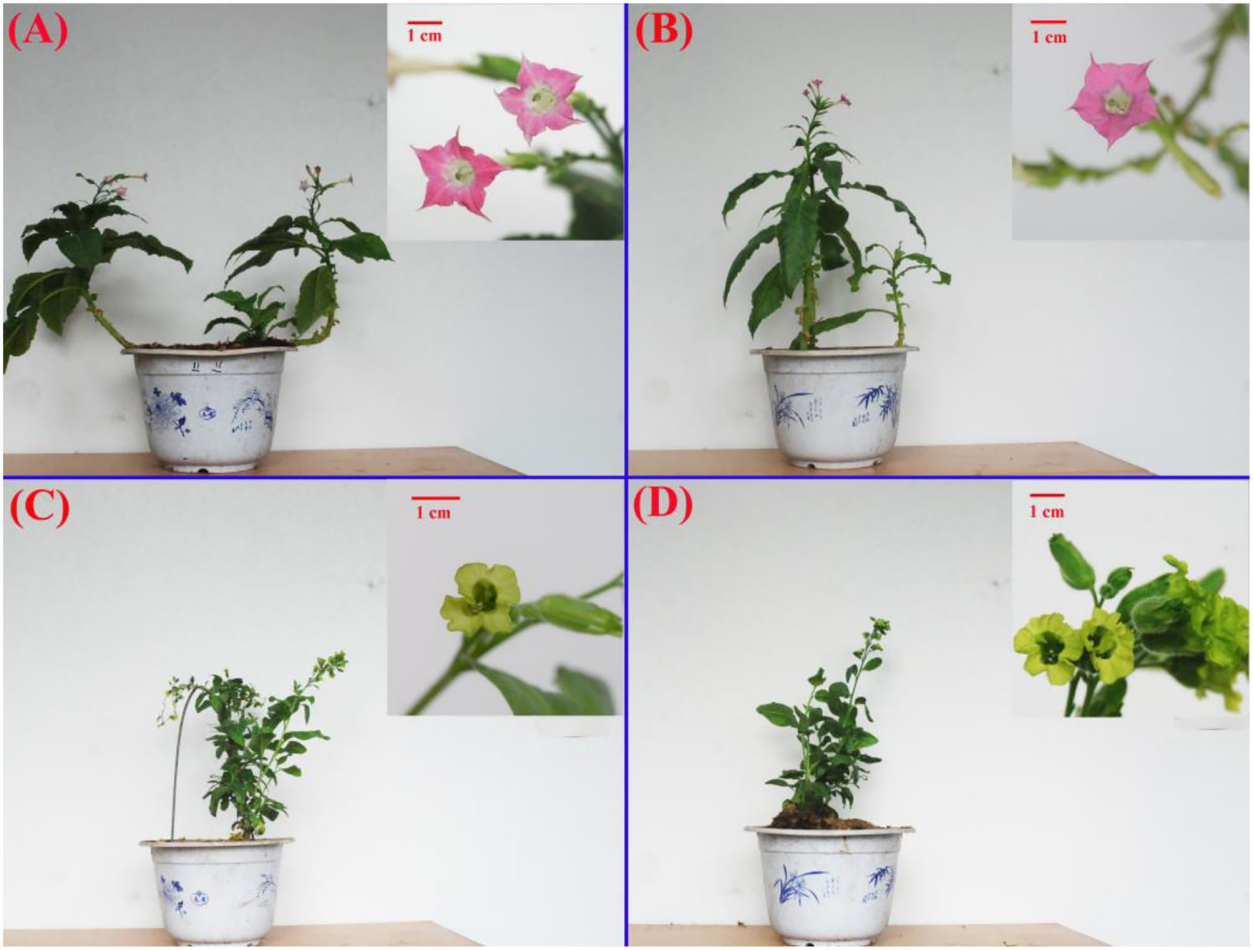
Morphology of the four *Nicotiana* cultivars. **(A)** and **(B)** *Nicotiana tabacum* cultivars RFA (No-2146) and RFB (Xiangyan-3) and **(C)** and **(D)** *Nicotiana rustica* cultivars YFA (Laolaihuang) and YFB (Xiaoyelanhua). The small patches placed onto each board at the right top were featured flowers, correspondingly. The red bar shows 1 cm.

The flower color and petal shape are vital traits to characterize *Nicotiana* germplasms. RFA (No-2146) and RFB (Xiangyan-3) were typical genera of *N. tabacum*, which both showed red (pink) flowers (shown in Figure 1). Parallelly, YFA (Laolaihuang) and YFB (Xiaoyelanhua) were representative genera of *N. rustica*, delivering yellow (orange) flowers. The petals of RFA and RFB showed with sharp tips, and the shapes of petals involved in YFA and YFB presented round or curved edges. The flower of RFA and RFB exhibited an obvious pentagram from the quarter view, whereas YFA and YFB gave a specious ring. As a whole, RFA and RFB demonstrated the more extensive leaf areas, whereas the smaller leaf area appeared within YFA and YFB. Compared with *N. tabacum*, there were more secondary branches within *N. rustica*.

Features of ion chromatogram on flavonoids and carotenoids demonstrate that the APCI–MS/MS and ESI–MS/MS produced reliable and high-quality data. The extracted ion chromatogram exhibited independent and sharp peaks of flavonoids (Supplementary Figure 1-1A). The targeted ingredients were isolated successfully. Additionally, overlapping spectra of total ion chromatogram of quality control samples in positive ion mode indicated the isolations were reliable (Supplementary Figure 1-1B). The normalized area of ion intensity reflected the individual ingredient content (Supplementary Figure 1-1C). All these indicated that the LC–MS Assay System was working correctly, and the high-quality data were produced perfectly. Parallelly, the assay system involved in carotenoids demonstrated reliable circumstances (Supplementary Figure 1-2).

### Profiles of Components Involved in Flavonoids and Carotenoids Between the Species

To qualify the metabolites of flavonoids, 108 types of the standard solution were made involving the concentrations of gradients 0.01, 0.02, 0.05, 0.1, 0.5,1, 5, 10, 50, 100, 500, 1,000, 2,000, and 5,000 ng/ml. Corresponding quantitative signals of each concentration standard were obtained. The standard curves of different substances were drawn based on a horizontal coordinate of standard concentrations and longitudinal coordinates of peak area. The standard curve linear equations and related coefficients of the targeted substances were shown in Supplementary Table 1. All 108 coefficients *rs* were higher than 0.997. It is clear that the linear equations were reliable. Similarly, regarding carotenoids, the concentrations of gradients included 0.001, 0.005, 0.01, 0.05, 0.1, 0.5, 1, 5, 10, 50, 100, 250, and 400 μg/ml. The standard curves were drawn based on the horizontal coordinates involving the concentration ratios of external and internal standards, and the longitudinal coordinates in terms of the concentration ratios of the area ratio of external and internal standards. A total of 68 standard curve linear equations were obtained, whose coefficients *rs* were higher than 0.991. The *rs* indicated that the standard equations in terms of carotenoids were reliable, although the values of *r* were lower than those within flavonoids. From the results on the PCA analysis of all samples (Supplementary Figure 2), it is illustrated that the samples belonging to the same cultivar had good reproducibility of these samples as given flocking together. Overall, cultivars RFA and RFB exhibited better reproducibility than that between YFA and YFB.

**Figure 2:**
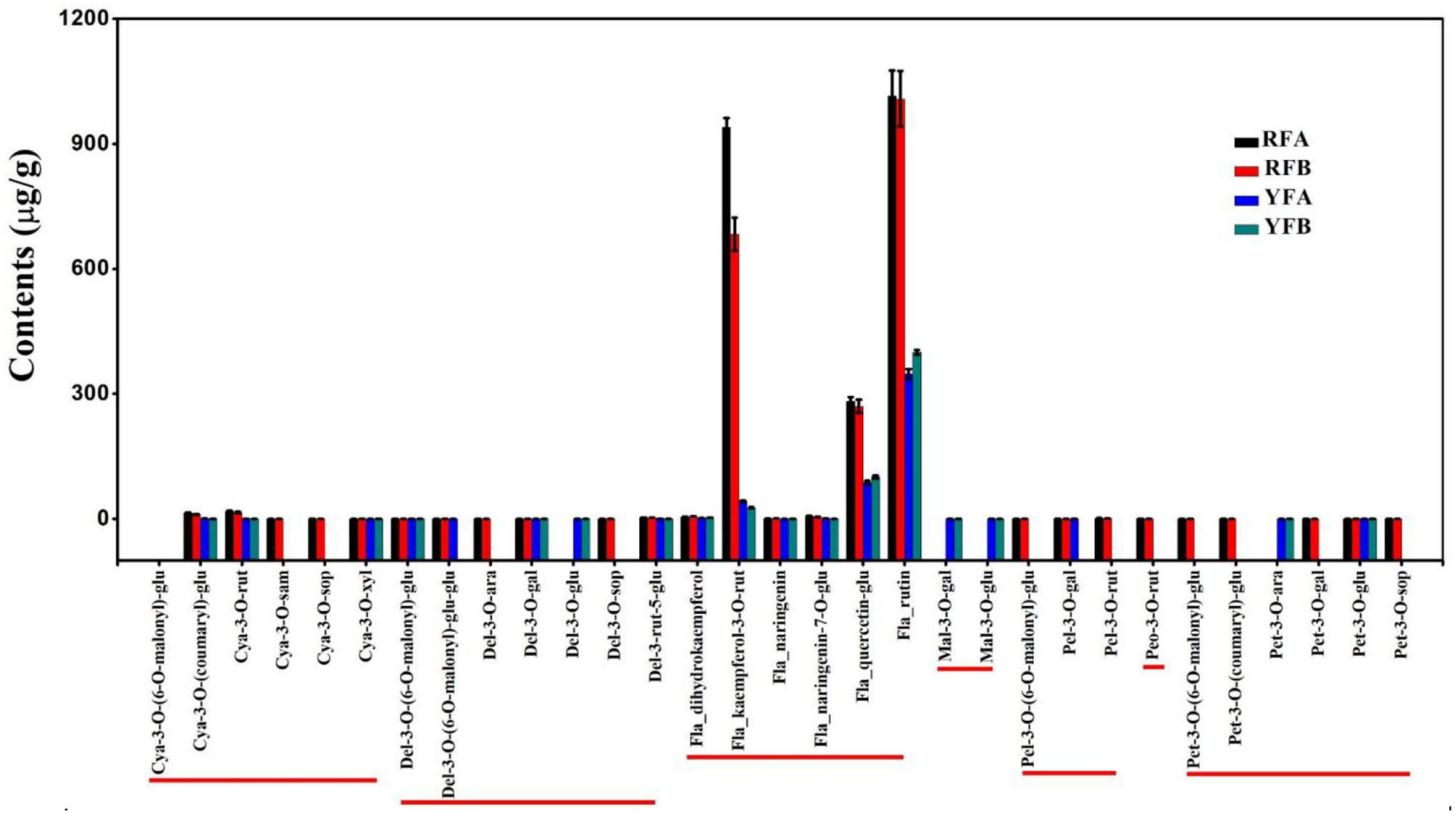
Histogram of flavonoids detected in the four cultivars. RFA and RFB denote the *Nicotiana tabacum* cultivars No-2146 and Xiangyan-3, respectively. YFA and YFB denotes the *Nicotiana rustica* cultivars Laolaihuang and Xiaoyelanhua, respectively. Cya, Del, Fla, Mal, Pel, Peo, and Pet are abbreviated from cyanidin, delphinidin, flavonoid, malvidin, pelnidin, peonidin, and petunidin, respectively.

Among 12 samples involved in the four species, 31 metabolites in flavonoids were identified and quantified as Figure 2. These metabolites are of seven types of anthocyanins, including cyanidin, delphinidin, malvidin, pelnidin, peonidin, and petunidin. Meanwhile, there were other specific flavonoids such as kaempferol, naringenin, and quercetin. In total, 13 substances were quantified in all four cultivars. As a whole, metabolites belonging to anthocyanins showed lower levels than those of other flavonoids. Remarkably, kaempferol-3-o-rut, quercetin-glu, and rutin contents were profoundly higher than those of the remaining metabolites. In the cultivars, RFA and RFB, the values of kaempferol-3-o-rut were as high as 939.84 and 683.11 μg/g, respectively. However, kaempferol-3-o-rut contents were 42.30 and 26.67 μg/g within YFA and YFB, respectively. Regarding quercetin-glu, the values were 282.16, 270.20, 87.51, and 100.30 μg/g in the above cultivars, respectively. Additionally, the means of rutin involved in the four cultivars were 1,014.90, 1,008.40, 347.69, and 399.78 μg/g. Overall, the values of metabolites in terms of anthocyanins fell into the range from 0.03 to 18.39 μg/g in all samples, which showed low levels. In anthocyanins, more sub-types of metabolites including cyanidin, delphinidin, pelnidin, peonidin and petunidin, except for malvidin, were identified in cultivars RFA and RFB, successfully. Overall, cultivars RFA and RFB presented more and higher accumulation of flavonoids than cultivars YFA and YFB.

**Figure 3:**
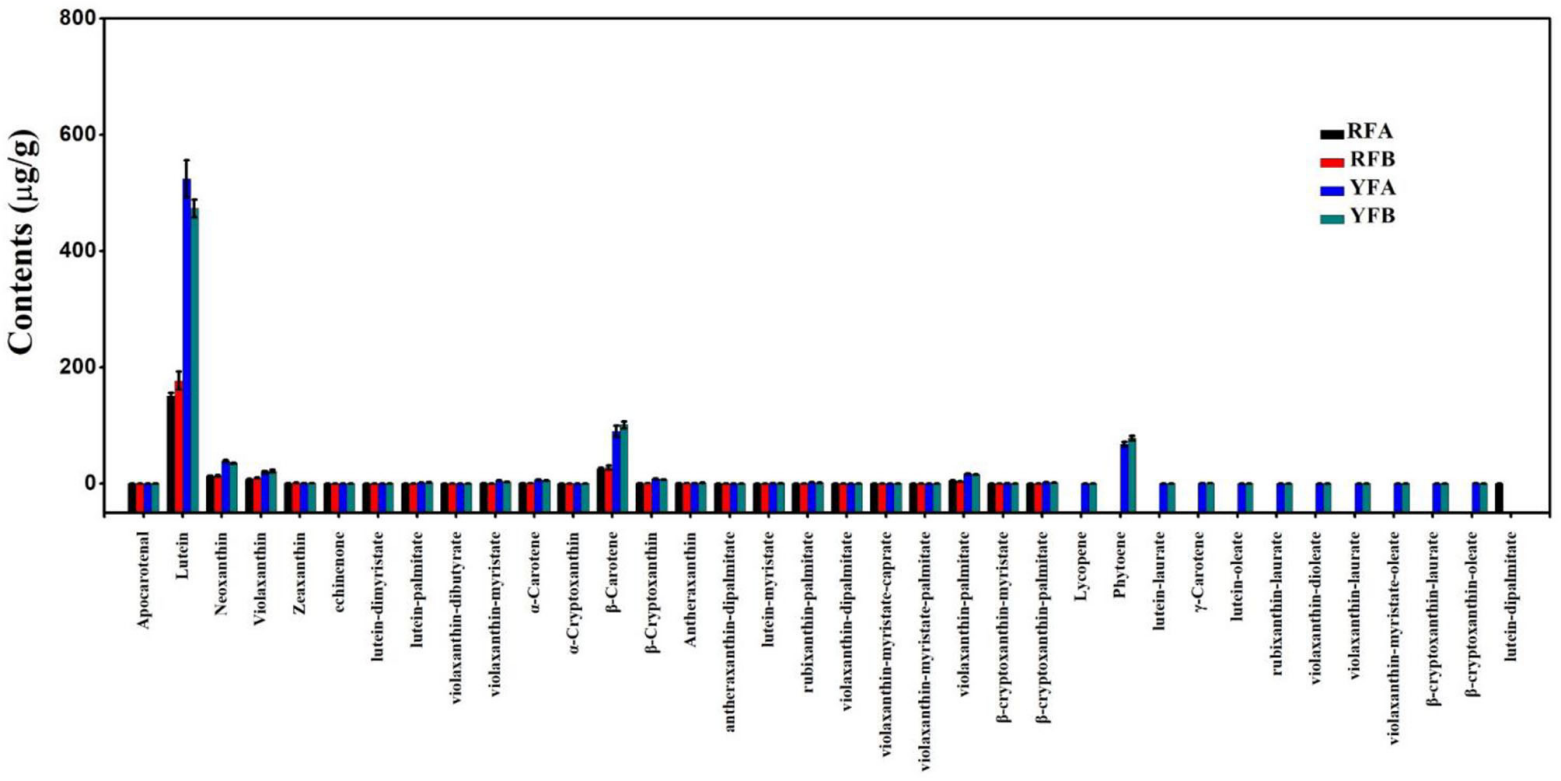
Histogram of carotenoids detected in the four cultivars. RFA and RFB denote the *N. tabacum* cultivars No-2146 and Xiangyan-3, respectively. YFA and YFB denotes the *Nicotiana rustica* cultivars Laolaihuang and Xiaoyelanhua, respectively.

Similarly, 36 carotenoids were finally identified from the four cultivars, shown in Figure 3. A total of 24 metabolites were quantified entirely in all four cultivars. Significantly, the lutein and β-carotene demonstrated comparatively super-higher values. Within the cultivars, FRA, FRB, YFA, and YFB, the means of lutein were 151.00, 177.00, 524.00, and 473.00 μg/g, respectively. Moreover, the contents of β-carotene in related cultivars were 25.60 27.60, 90.10, and 101.00 μg/g. A total of 11 carotenoids were successfully identified in the flowers of YFA and YFB, while there were no values in those of RFA and RFB. They were lycopene, phytoene, lutein–laurate, γ-carotene, lutein–oleate, rubixanthin–laurate, violaxanthin–dioleate, violaxanthin–laurate, violaxanthin–myristate–oleate, β-cryptoxanthin–laurate, and β-cryptoxanthin–oleate. Moreover, the phytoene presented higher values with 67.90 and 78.10 μg/g in the cultivars YFA and YFB, respectively. As a whole, more types and higher levels of carotenoids were identified from cultivars YFA and YFB, compared with those in cultivars RFA and RFB.

### Hierarchical Cluster Analysis Among Species

**Figure 4:**
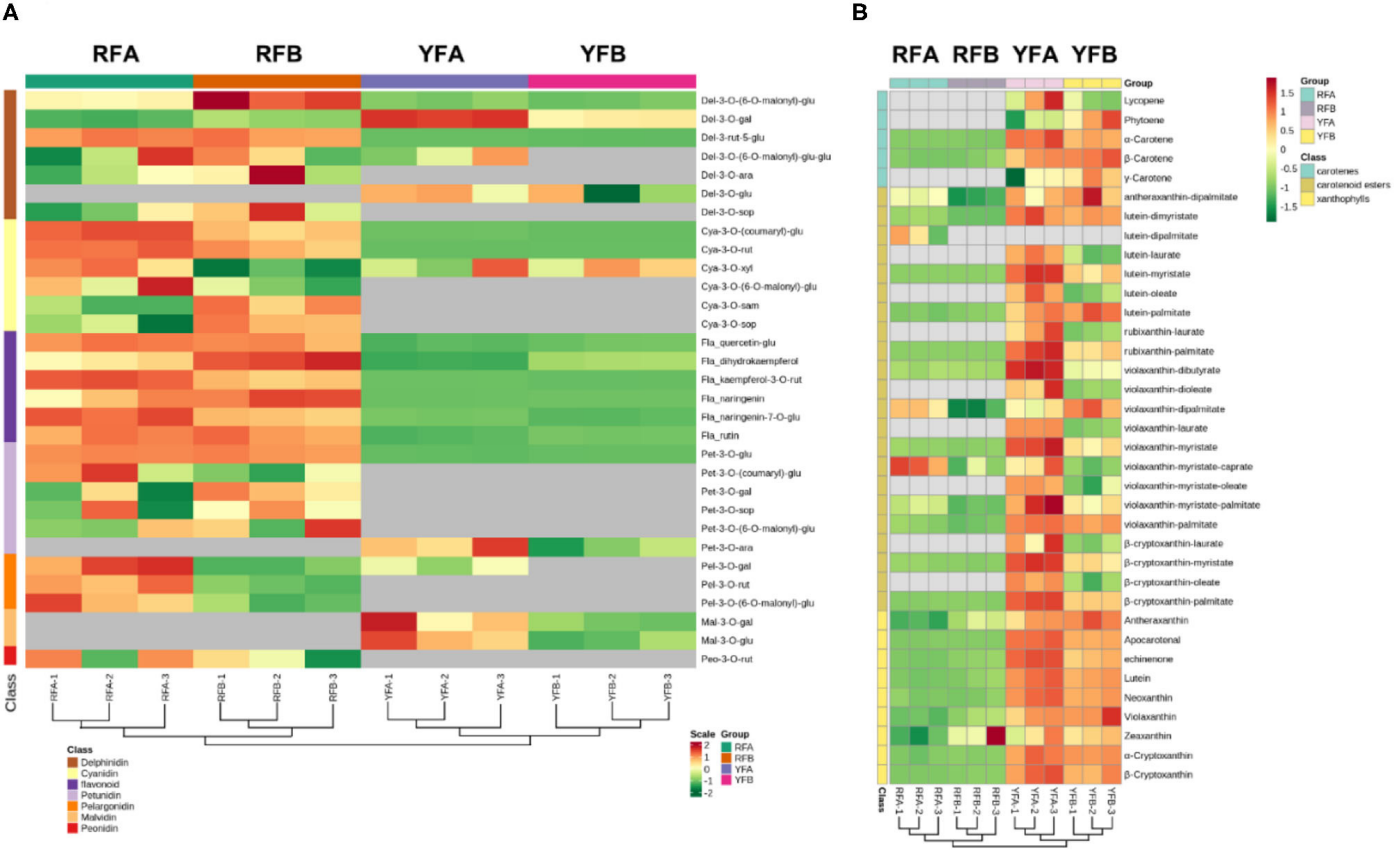
Cluster analysis on the samples and metabolites depending on classes. **(A)** and **(B)** demonstrate features of clusters in terms of flavonoids and carotenoids. RFA and RFB denote the *Nicotiana tabacum* cultivars No-2146 and Xiangyan-3, respectively. YFA and YFB represents the *Nicotiana rustica* cultivars Laolaihuang and Xiaoyelanhua, respectively.

A total of 31 flavonoids and 36 carotenoids in 12 samples were hierarchically clustered, as shown in Figure 4. The samples exhibited the same topological structures involving both flavonoids and carotenoids. In detail, replicates FRA-1 and FRA-2, FRB-1 and RFB-2, YFA-1 and YFA-2, plus YFB-1 and YFB-2 showed intimate hierarchical relationships. The replicates belonging to the same cultivars were clustered together. RFA and RFB were close to each other in the four cultivars, as the same case appeared on YFA and YFB. The two groups of species exhibited the same hierarchical level, that is, the metabolisms rarely diverged between the species RFA and RFB or YFA and YFB based on flavonoids and carotenoids. However, the group involving RFA and RFB was differentiated from the YFA and YFB in terms of flavonoids and carotenoids. Metabolites that appeared to have a higher and varied accumulation between the two groups can be screened out as markers, under the premise of consistent accumulation in the individual group. Regarding flavonoids, Del-3-rut-5-glu showed a higher level in the species of RFA and RFB, yet a lower level in YFA and YFB. Consequently, it can be chosen as a marker. Similarly, another marker belonging to anthocyanin was Pet-3-o-glu. Furthermore, the other flavonoid markers included quercetin-glu, dihydrokaempferol, kaempferol-3-*o*-rut, naringenin, naringenin-7-*o*-glu, and rutin. As shown in Figure 4B, the carotenoids are of three types, namely, carotenes, carotenoid esters, and xanthophylls. The metabolites involved in the sort of xanthophylls demonstrated more uniform and significant accumulation between two species groups. Overall, the markers in terms of carotenoids included α-carotene, β-carotene, lutein–dimyristate, lutein—palmitate, violaxanthin–palmitate, apocarotenal, echinenone, lutein, neoxanthin, violaxanthin, α-cryptoxanthin, and β-cryptoxanthin.

### Comparative Analysis of the Accumulation of Metabolites

**Figure 5:**
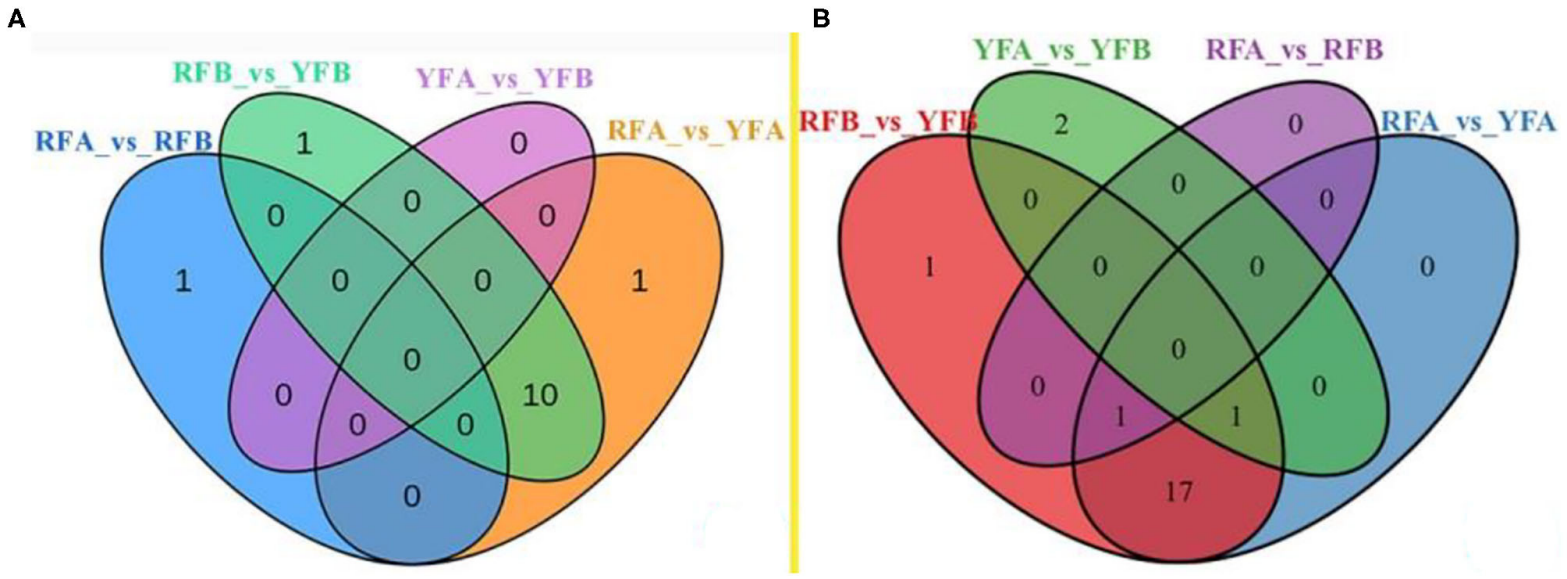
Venn diagram on the overlapping and species-specific differential metabolites. **(A)** and **(B)** were involved in flavonoids and carotenoids, respectively. RFA and RFB, and YFA and YFB represent *Nicotiana tabacum* cultivars No-2146 and Xiangyan-3, and *Nicotiana rustica* cultivars Laolaihuang and Xiaoyelanhua, respectively.

Differential metabolites screened in cultivars are shown in Supplementary Figure 3. The comparative analysis of flavonoids and carotenoids was established as a Venn diagram (Figure 5). Regarding flavonoids, only a single metabolite (Pel-3-O-rut and lutein–dimyristate, respectively) exhibited a different accumulation between cultivars RFA and RFB. Similarly, there was no significant enrichment of metabolite between YFA and YFB. Parentally, there was rare variation between the cultivars in the same species. On the contrary, 11 metabolites in terms of flavonoids had a varied accumulation between RFB and YFB. Correspondingly, the numbers of metabolites variously enriched between RFA and YFA were 11, too. A total of 10 metabolites were diverging between RFA_*vs*_YFA and RFB_*vs*_YFB. The data illustrated that the flavonoids of cultivars belonging to different species were various. Only one carotenoid presented the same accumulation between cultivar RFA and RFB. Three carotenoids were significantly enriched between cultivars YFA and YFB. Analogously, there were fewer carotenoids that can be accumulated between the cultivars belonging to the same group. About 19 and 20 differentiated abundant carotenoids appeared within RFA_*vs*_YFA and RFB_*vs*_YFB, respectively. Seven essential carotenoids were significantly accumulated between the species.

### Pathways of Flavonoid and Carotenoid Synthesis

**Figure 6:**
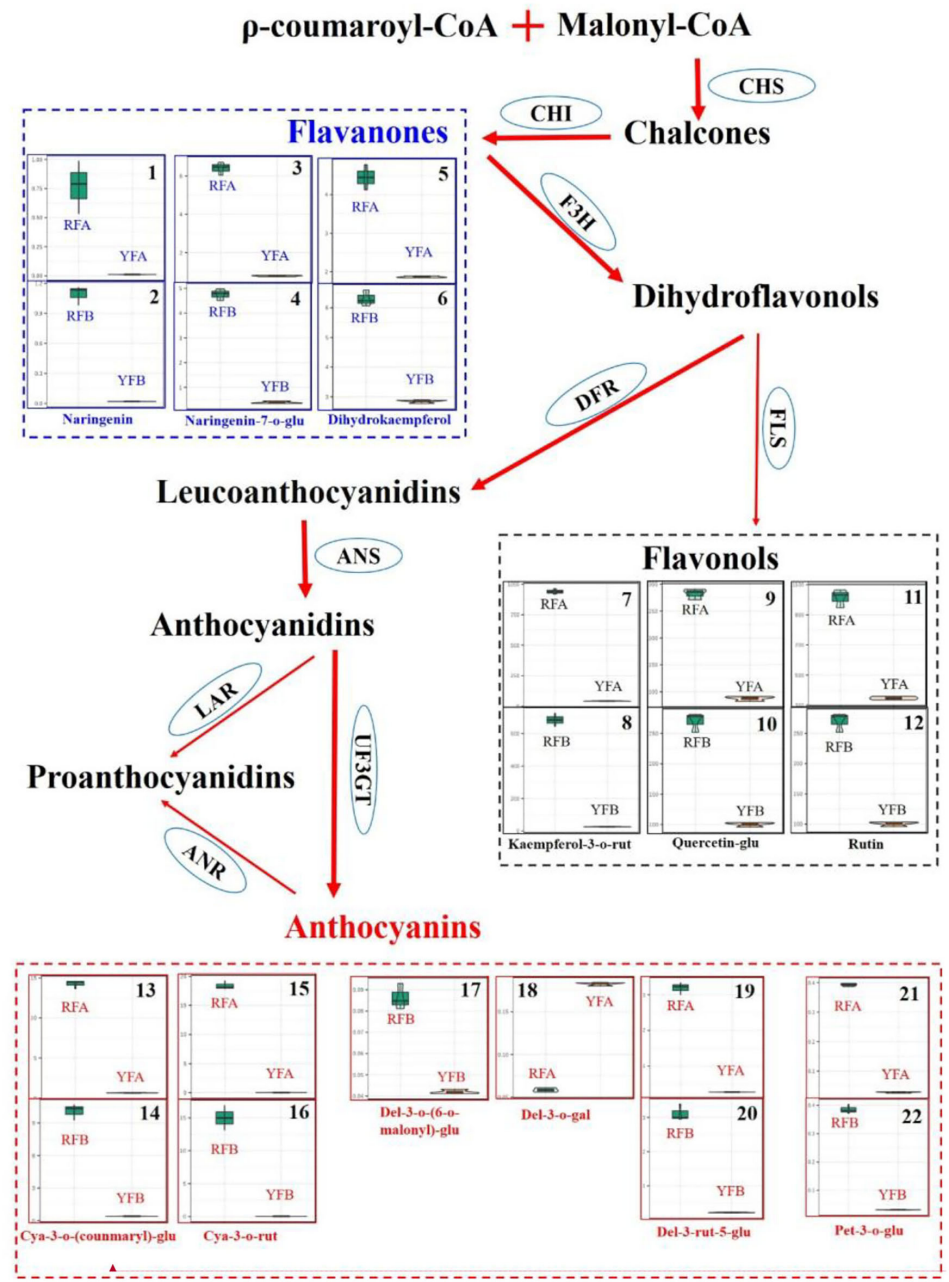
Differentiate metabolisms of flavonoids synthesis between *Nicotiana tabacum* and *Nicotiana rustica*. Each square involving violin plots represents a metabolite. The metabolites were compared between cultivars RFA (No-2146) and YFA (Laolaihuang) or RFB (Xiangyan-3) and YFB (Xiaoyelanhua). The squares adhering without separation represent the same metabolites. The ellipses denote enzymes. CHS, Chalcone synthase; CHI, Chalcone isomerase; F3H, Flavanone 3-hydroxylase; FLS, Flavonol synthase; DFR, Dihydroflavonol reductase; ANS, Anthocyanidin synthase; UF3GT, Flavonoid 3-o-glucosyltransferase; LAR, Leucoanthocyanidin reductase; ANR, Anthocyanidin reductase.

Initiated from ρ-coumaroyl-CoA and malonyl-CoA, flavonoids were synthesized, involving key intermediates chalcones, flavanones, dihydroflavonols, and leucoanthocyanidins. Meanwhile, flavonols were derived from the branch of dihydroflavonols (Figure 6). Three classes of flavonoids, including cyanidins, delphinidins, and petunidins, were mapped onto the pathway. cya-3-*o*-(coumaryl)-glu and cya-3-rut represented exactly similar features in violin plot among four cultivars, and they showed the close higher and lower values within the same species. In other words, cyanidins quickly accumulated in the cultivars RFA and RFB, compared with YFA and YFB. Because of pet-3-*o*-glu, it seemed that petunidins kept in line with cyanidin involved in species in the anthocyanins synthesis pathway of tobacco. The delphinidins del-3-*o*-(6-*o*-malony)-glu and del-3-rut-5-glu showed similar profiles with other above-mentioned cyanidins in this pathway. However, del-3-*o*-gal demonstrated the specific values in cultivars RFA and YFA, as higher accumulated in YFA, nor RFA. Three flavanones, naringenin, naringenin-7-*o*-glu, and dihydrokaempferol, were mapped on the pathway. Naringenin was rarely enriched in cultivars YFA and YFB, while those in RFA and RFB showed higher values and variations. Compared with that in RFA, naringenin in sample replicates of RFB presented closer maximum and minimum. Naringenin-7-*o*-glu were highly enriched in the cultivars RFA and RFB, while strictly lowly accumulated in YFA and YFB. Three flavonols involving kaempferol-3-*o*-rut, quercetin-glu, and rutin consistently demonstrated accumulation in all cultivars. Again, the higher and lower values were detected in the group of RFA and RFB and the group of YFA and YFB, respectively.

In the carotenoid synthesis pathway, two main branches involving δ-carotene and γ-carotene have diverged from lycopene. The δ-carotene routine included α-carotene, zeinoxanthin, lutein, and 5, 6-epoxylutein. Correspondingly, the γ-carotene routine involved β-carotene, zeaxanthin, antheraxanthin, violaxanthin, and neoxanthin. Totally, eight carotenoids were mapped onto the carotenoid synthesis pathway. As a whole, the carotenoids were highly synthesized in the cultivars YFA and YFB. δ-Carotene was enriched more than five times in the group of YFA and YFB, compared with those in the group of species RFA and RFB. Four kinds of luteins, including lutein, lutein–dimyristate, lutein–myristate, and lutein—palmitate, were detected in the pathway. These four types of lutein showed more than double accumulation in the cultivars YFA and YFB, compared with RYA and RYB. Regarding another branch, there was no apparent increase or decrease in γ-carotene between the species RFA and YFA, given the bars of the replicates. However, their next-step derivative β-carotene presented more than two times increase in the cultivars YFA and YFB, compared with those in the group of species RFA and RFB. Interestingly, the synthesis of zeaxanthin showed very close values in the four cultivars, which agreed with γ-carotene. Two types of antheraxanthin were detected in this pathway, and they demonstrated slight decline in YFA and YFB, compared with those in RFA and RFB. Seven types of violaxanthin were mapped onto the routine of γ-carotene. Except for violaxanthin–dipalmitate and violaxanthin–myristate–caprate, apparent increases were observed in the remaining five types of violaxanthin in terms of the cultivars YFA and YFB. Regarding neoxanthin, the profiles generally kept agreeing with other carotenoids in this pathway.

## Discussion

### The Red Color of Flowers That Appeared in Species *N. tabacum* Are Mainly Contributed by Flavonoids

In the study, the flavonoids were ultra-highly accumulated in the red flowers of species *N. tabacum*, which involved cultivars RFA (No-2146) and RFB (Xiangyan-3). On the contrary, the flavonoids were rarely enriched in the yellow flowers of species *N. rustica*, which included cultivars YFA (Laolaihuang) and YFB (Xiaoyelanhua). Flavonoids are the most common pigment group and present a broad spectrum of colors from pale yellow to blue-pure, and it is reported the most vital pigments in petals (Zhao and Tao, 2015). Flavonoids were detected in various plant flowers, such as groundcover rose, herbaceous peony, dahlia, violet, and so on (Schmitzer, 2010; Tatsuzawa et al., 2012; Thill et al., 2012; Zhao et al., 2012). Chalcone isomerase (CHI, EC 5.5.1.6) is a vital enzyme in the pathway of plant flavonoids biosynthesis, as it catalyzes the cyclization of chalcones and consequently produces flavanones. It is a practical approach to change the composition of flavonoids *via* downregulating or enhancing the expression of *CHI*. For example, tomato flavonols contents were increased after the chi was overexpressed (Muir et al., 2001). Moreover, the flower colors could be modified *via* the changes of flavonoids based on other vital enzymes such as chalcones synthase (CHS) (Mol et al., 1999; Forkmann and Martens, 2001). In *Nicotiana*, the altered composition of flavonoids resulted in the change of flower color. The transgenic tobacco (*Nicotiana tabacum* L. cv. SR1) by RNAi transformation causes the suppression of CHI and consequently disappearance of the red color of flowers (Nishihara et al., 2005). This study systematically compared three main classes of flavonoids, regarding flavanones, flavonols, and anthocyanins, in *Nicotiana* species. The red flowers contained higher these three classes of flavonoids, whereas the yellow flowers did not accumulate much on these three classes. The compositions of flavonoids posed crucial effects on the formation of flower color. In other plants, the compositions of flavonoids could be changed in the same species. For instance, the pink flowers contained anthocyanins, flavones, and flavonols in chrysanthemums, while the white flower had flavones and flavonols merely (Chen et al., 2012). In *Lycoris longituba*, less common anthocyanins were identified among the various-colored flowers (He et al., 2011). However, there were no apparent alternatives of flavonoids between cultivars belonging to the same *Nicotiana* species in our studies. The cultivars in the same species were probably with close genetic background. It was considered that anthocyanin belongs to the red series and determines the flower colors ranging from pink to blue. In contrast, the other flavonoids belong to the pure yellow series, where flavones, flavanones, and flavonols are light yellow and even close to colorless, and chalcones and aurones are deep yellows (Zhao and Tao, 2015). Our results did not strictly agree with this and inferred they were complex processes within the coloration determined by flavonoids. Jiao et al. (2020) discovered some anthocyanins' down-accumulation in white flower mutants of *N. tabacum*, compared with those in the red flowers of wild type, of which Cya-3-*o*-glu and Pel-3-*o*-glu in our research had an analogous accumulation in the red nor yellow flowers.

### The Yellow Color of Flowers Appeared in Species *N. rustica* With Higher Carotenoid Accumulation

**Figure 7:**
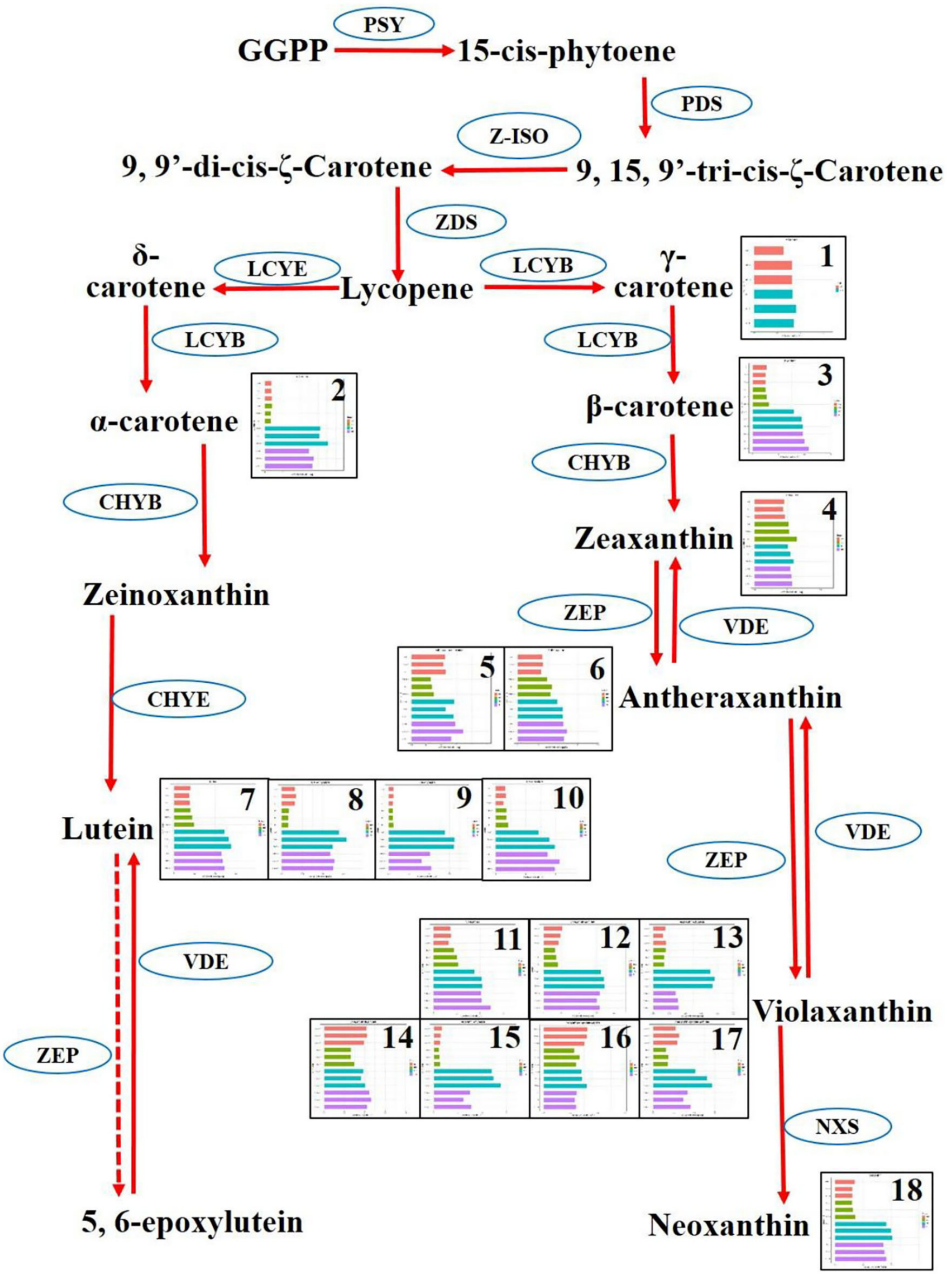
Differentiate metabolisms of carotenoid synthesis between *Nicotiana tabacum* and *Nicotiana rustica*. Each square represented a metabolite. The replicates and four cultivars were exhibited in the squares. The red, yellow, cyan, and pink denote RFA (No-2146), RFB (Xiangyan-3), YFA (Laolaihuang), and YFB (Xiaoyelanhua), respectively. The squares were numbered and named as 1. γ-Carotene, 2. α-Carotene, 3. β-Carotene, 4. Zeaxanthin, 5. Antheraxanthin–dipalmitate, 6. Antheraxanthin, 7. Lutein, 8. Lutein–dimyristate, 9. Lutein–myristate, 10. Lutein—palmitate, 11. Violaxanthin, 12. Violaxanthin–palmitate, 13. Violaxanthin–dibutyrate, 14. Violaxanthin–dipalmitate, 15. Violaxanthin–myristate, 16. Violaxanthin-myristate-caprate, 17. Violaxanthin–myristate-palmitate, 18. Neoxanthin. The ellipses denote enzymes. PSY, Phytoene synthase; PDS, Phytoene desaturase; Z-ISO, ζ-carotene isomerase; ZDS, ζ-carotene desaturase; LCYE, Epsilon cyclase; LCYB, β-cyclase; ZEP, Zeaxanthin epoxidase; VDE, Violaxanthin de-epoxidase; NXS, Neoxanthin synthase.

**Figure 8:**
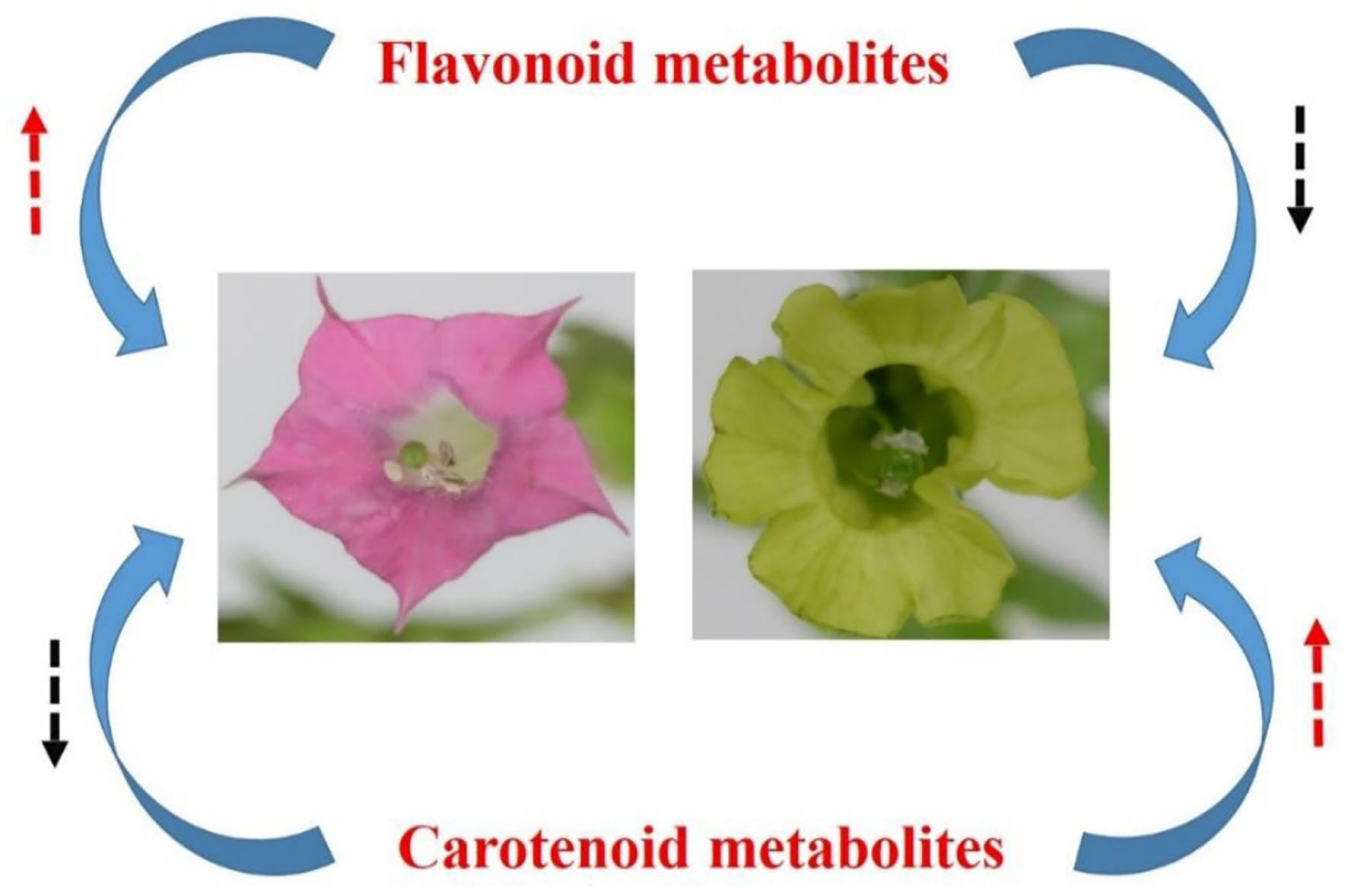
Hypothetical metabolic model on the divergence of flower color between *N. tabacum* and *N. rustica*. The red and black dash arrows denote accumulation and decline.

Carotenoids were more enriched in yellow flowers, in species *N. rustica*, including cultivars YFA (Laolaihuang) and YFB (Xiaoyelanhua). However, there was less carotenoid accumulation in the red flowers of species *N. tabacum* involving RFA (No-2146) and RFB (Xiangyan-3). In plants, carotenoids exist in photosynthetic and non-photosynthetic organs (Yuan et al., 2015). Mainly, carotenoids provide bright colors in non-photosynthetic tissues. The compositions of carotenoids are not the same among species, despite the flower representing the close color in the petals (Zhao and Tao, 2015). In the yellow flowers of species *N. rustica*, α-carotenes, luteins, β-carotenes, zeaxanthins, antheraxanthins, violaxanthins, and neoxanthins were much more enriched. In the carotenoid synthesis (shown in Figure 7), ζ-carotene is yellow, and lycopene is red. Lycopene cyclase produces carotenoids with cyclic terminal end groups such as α-carotene and β-carotene, which appeared with orange-yellow and yellow colors, respectively (Maoka, 2020). In *Osmanthus*, minor accumulation of α-carotene resulted in golden yellow, while significant enrichment of β-carotene produced butter yellow (Han et al., 2014). Regarding luteins and their epoxides, the higher accumulation leads the color ranging from yellow to orange in varieties such as *Marigold* and *Chrysanthemum* (Kishimoto and Ohmiya, 2006; Zhu et al., 2014). Minor accumulation of luteins showed yellow and orange flowers in species *Oncidium* and *Osmanthus* (Chiou et al., 2010; Han et al., 2014). Minor antheraxanthin accumulated in the flower of *Lili* var. Saija and Tiger showed red color (Yamagishi et al., 2010; Jekni et al., 2012). However, the flowers were yellow in *Lily* var. Connecticut King, when antheraxanthins were detected combing with violaxianthins and luteins simultaneously (Yamagishi et al., 2010). The plant flowers contain a single type of carotenoids such as antheraxanthins rarely, and thereby flowers enriched with carotenoids usually show yellow color. It is reported that only violaxanthins concentrated profoundly in *Oncidium* var. Gower Ramsey and subsequently presented yellow flowers. Additionally, the combing accumulation on violaxanthins and β-carotene generated orange flowers in the *Oncidium* var. Sunkist (Chiou et al., 2010). In general, carotenoids lead to yellow flowers in plants, especially those containing more than two classes. In *N. rustica*, the yellow-color of the flowers was probably due to the appearance of multiple types of carotenoids (shown in Figure 8).

### Exploring Novel Metabolic Marker–Involved Metabolites Is an Effective Strategy to Identify Traits

The metabolome allowed exploration on metabolic biomarkers, besides dissecting the metabolic mechanisms underlying divergence between red and yellow flowers of *Nicotiana*. Some specifically distinct and ultra-higher accumulated metabolites were screened out in this study, such as kaempferol-3-*o*-rut, quercetin-glu, rutin, lutein, β-carotene (Figures 2, 3). Generally, the biomarker should be employed in populations, and it should not be influenced by different environments in plant breeding (Steinfath et al., 2010). Indeed, typical marker-assisted selection has been proved to be very successful for many diploid crops. However, applying genetic markers is still problematic for species with a complex polyploid genome, which is prevailing in many crop plants. Another difficulty for genetic markers is that they fail to apply for polygenic traits directly. A batch of metabolites, relating to tobacco flower coloration were screened out for biomarkers in our study. Besides the traditional biomarker selection, these metabolic biomarkers must enrich the means to improve tobaccos.

## Conclusions

The main findings were concluded as follows:

- A total of 31 flavonoids and 36 carotenoids were identified in all four cultivars involved in *N. tabacum* and *N. rustica*.
- Flavonoids and carotenoids tended to concentrate in the red flowers (*N. tabacum*) and yellow flowers (*N. rustica*), respectively.
- Eight flavonoids and 12 carotenoids were primarily screened out for metabolic biomarkers, such as the robust biomarker involving kaempferol-3-*o*-rut, quercetin-glu, rutin, lutein, and β-carotene.

## Data Availability Statement

The original contributions presented in the study are included in the article/Supplementary Material, further inquiries can be directed to the corresponding author.

## Author Contributions

QX and LM designed the study. QX and LL grew the plants. YZ, GC, RH, LW, and ZD performed the qualifying and quantifying on the metabolism involving anthocyanin and carotenoid. XZ, ZD, and LM collected and analyzed the data. RH, LL, and LW contributed to the illustration. All authors contributed to the manuscript writing and approved the submitted version.

## Funding

This work was funded by the Project of Yongzhou Tobacco Monopoly Bureau with grant number *yzyc2019KJ02*, the China Postdoctoral Science Foundation with grant number *2021M690633*, and the Natural Science Foundation of Hubei Province, China, with grant number *2021CFB002*.

## Conflict of Interest

QX, RH, and ZD were employed by Yongzhou Tobacco Monopoly Bureau of Hunan. The remaining authors declare that the research was conducted in the absence of any commercial or financial relationships that could be construed as a potential conflict of interest.

## Publisher's Note

All claims expressed in this article are solely those of the authors and do not necessarily represent those of their affiliated organizations, or those of the publisher, the editors and the reviewers. Any product that may be evaluated in this article, or claim that may be made by its manufacturer, is not guaranteed or endorsed by the publisher.

## Supplementary Material

Supplementary Figure 1-1 Features of ion chromatogram on anthocyanins. **(A)** Extracted ion chromatogram, **(B)** overlapping spectra of TIC of QC samples in positive ion mode, and **(C)** normalized area of ion intensity. TIC, total ion chromatogram; QC, quality control.

Supplementary Figure 1-2 Features of ion chromatogram on carotenoids. **(A)** Extracted ion chromatogram, **(B)** overlapping spectra of TIC of QC samples in positive ion mode, and **(C)** normalized area of ion intensity. TIC, total ion chromatogram; QC, quality control.

Supplementary Figure 2 Principal component analysis on 12 samples based on all metabolites.

Supplementary Table 1 Equation of standard curve involved in anthocyanins, where *r* represents the coefficient of correlation.

Supplementary Table 2 Equation of standard curve involved in carotenoids, where *r* represents the coefficient of correlation.

Supplementary Table 3 Differential metabolites among cultivars and involved Venn diagrams.
