# Supplementary figures and images for: A Comparative Study of Flavonoids and Carotenoids Revealed Metabolite Responses for Various Flower Colorations Between Nicotiana tabacum L. and Nicotiana rustica L
Source: Front Plant Sci. 2022 Apr 25;13:828042. doi: 10.3389/fpls.2022.828042 (PMC9083207; doi:10.3389/fpls.2022.828042)

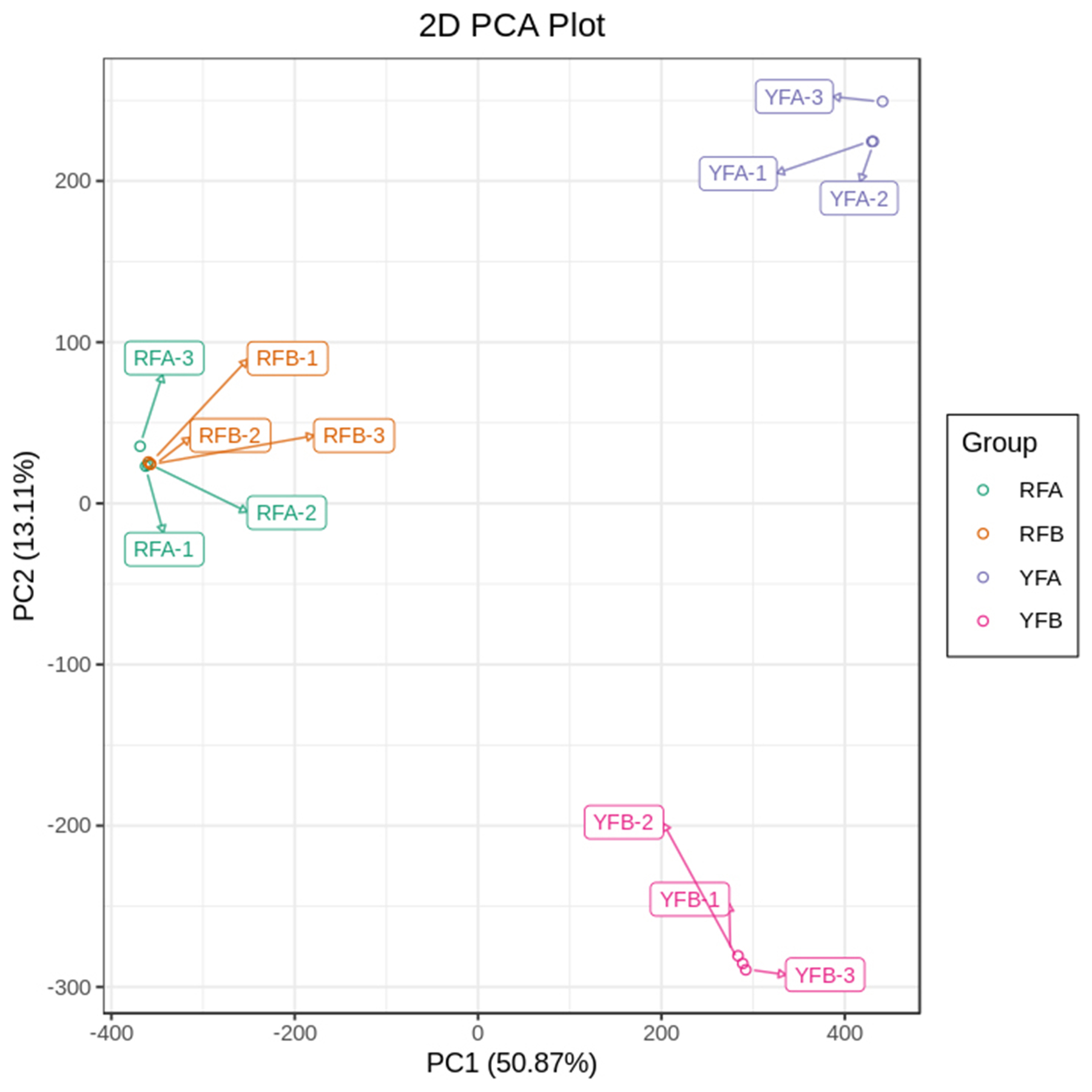

Supplement: Supplementary Figure 2 — Principal component analysis on 12 samples based on all metabolites. [file Image_3.jpg]
